# Prognostic Impact of Postoperative Recurrence in Patients With Epidermal Growth Factor Receptor–Positive Non‐Small Cell Lung Cancer

**DOI:** 10.1002/cnr2.70004

**Published:** 2024-09-08

**Authors:** Meiko Morita, Akira Ono, Motoki Sekikawa, Kosei Doshita, Keita Miura, Hiroaki Kodama, Michitoshi Yabe, Noboru Morikawa, Yuko Iida, Nobuaki Mamesaya, Haruki Kobayashi, Ryo Ko, Kazushige Wakuda, Hirotsugu Kenmotsu, Tateaki Naito, Haruyasu Murakami, Mitsuhiro Isaka, Yasuhisa Ohde, Toshiaki Takahashi

**Affiliations:** ^1^ Division of Thoracic Oncology Shizuoka Cancer Center Shizuoka Japan; ^2^ Division of Thoracic Surgery Shizuoka Cancer Center Shizuoka Japan

**Keywords:** EGFR, non‐small cell lung cancer, oligometastasis, postoperative recurrence, prognostic factors

## Abstract

**Background:**

Mutations in the epidermal growth factor receptor (*EGFR*) gene are the most common targetable gene alterations in non‐small cell lung cancer (NSCLC). In Japan, approximately 40% of patients who undergo surgical resection for non‐squamous NSCLC have *EGFR* mutations. However, no long‐term studies have been conducted including a large number of *EGFR*‐positive NSCLC patients with postoperative recurrence (PR).

**Methods:**

We conducted a retrospective observational study of the data of *EGFR*‐positive NSCLC patients with PR who had undergone surgery at the Shizuoka Cancer Center between October 2002 and November 2017. We evaluated post‐recurrence overall survival (PRS) and postoperative overall survival (POS) using the Kaplan–Meier method and identify any associations between the clinical variables at recurrence and PRS using univariate and multivariate analysis.

**Results:**

We enrolled 162 patients. The median observation time for PRS was 4.95 years (range, 0.82–13.25) and POS was 5.81 years (range, 2.84–16.71). The median PRS was 5.17 years (95% confidence interval [CI], 3.90–5.61) and POS was 7.07 years (95% CI, 5.88–8.01). Univariate analysis identified male sex (median PRS: 3.32 vs. 5.39 years; *p* < 0.05), bone metastasis (median PRS: 2.43 vs. 5.33 years; *p* < 0.05), and central nervous system (CNS) metastasis (median PRS: 3.05 vs. 5.39 years; *p* < 0.05) and multivariate analysis identified bone metastasis (hazard ratio [HR], 2.01; 95% CI, 1.23–3.28; *p* < 0.05) and CNS metastasis (HR, 1.84; 95% CI, 1.14–2.98; *p* < 0.05) as poor prognostic factors. The pattern of recurrence (oligo vs. non‐oligo recurrence) was not a prognostic factor. Logistic regression analysis revealed the association between sex and the presence bone/CNS metastasis at recurrence.

**Conclusion:**

Our data may help visualize future prospects and determine the timing of osimertinib initiation. New treatment strategies need to be developed for patients with bone/CNS metastasis at the first recurrence.

## 1 Introduction

Mutations in the epidermal growth factor receptor (*EGFR*) gene are the most common targetable gene alterations in non‐small cell lung cancer (NSCLC). The same is true for postoperative recurrence (PR); approximately, 40% of patients who undergo surgical resection for non‐squamous NSCLC have *EGFR* mutations in Japan [1]. The efficacy of EGFR–tyrosine kinase inhibitors (EGFR–TKIs) for *EGFR*‐positive NSCLC is now well established [2]. Based on the findings of the FLAURA trial, which showed significantly prolonged progression‐free survival (PFS) and overall survival (OS) in the osimertinib arm compared to that in the standard treatment arm (median PFS: 18.9 months [95% confidence interval or CI, 15.2–21.4] vs. 10.2 months [95% CI, 9.6–11.1]; hazard ratio [HR], 0.46; *p* < 0.001; median OS: 38.6 months [95% CI, 34.5–41.8] vs. 31.8 months [95% CI, 26.6–36.0]; HR, 0.80; *p* < 0.05) [3, 4], osimertinib is now considered as the standard treatment agent for advanced *EGFR*‐positive NSCLC patients.

In the ADAURA trial, postoperative adjuvant osimertinib therapy improved the disease‐free survival (DFS) and OS in patients with *EGFR*‐positive NSCLC. The 5‐year survival rate was 88% (95% CI, 83–91)/78% (95% CI, 73–82) (HR, 0.49, *p* < 0.05) in the osimertinib/placebo group for patients with Stages IB–IIIA disease and 85% (95% CI, 79–89)/73% (95% CI, 66–78) (HR, 0.49, *p* < 0.05) for patients with Stages II–IIIA disease [5, 6]. Therefore, adjuvant osimertinib is now offered as an option after complete resection for patients with *EGFR*‐positive NSCLC. However, in clinical practice, patients often refuse the drug due to concerns about toxicity and treatment duration, or they do not wish to resume after withdrawal due to toxicity in the adjuvant setting.

Ko et al. compared the prognosis following treatment with gefitinib between *EGFR*‐positive NSCLC patients with Stage IV disease (119 patients) and *EGFR*‐positive NSCLC patients with PR (49 patients) and reported that the prognosis was better in patients with PR (median post‐recurrence overall survival [PRS]: 22.2 vs. 51.1 months; HR, 0.39; 95% CI, 0.22–0.66; *p* < 0.05) (median observation time [PRS], 24.6 months). In addition to PR, performance status and distant metastasis have also been reported as prognostic factors [7]; however, this study was limited by the insufficient number of patients and short observation time.

Although several reports have suggested male sex and the presence of brain metastasis as poor prognostic factors in NSCLC patients with PR [8, 9], no reports exist of prognostic factors identified in *EGFR*‐positive NSCLC patients with PR. Moreover, among NSCLC patients with PR, those with oligo recurrence have a better PRS than that of patients with non‐oligo recurrence (5‐year PRS: 32.9% vs. 9.9%; *p* < 0.05) [10]. We hypothesized that the presence or absence of CNS metastasis and the pattern of recurrence (oligo vs. non‐oligo recurrence) could be associated with survival in *EGFR*‐positive NSCLC patients with PR. Therefore, in order to be able to choose when to start EGFR–TKIs especially osimertinib, in this study, we evaluated OS in patients with long‐term follow‐up and investigated the impact of variables on the prognosis in *EGFR*‐positive NSCLC patients with PR.

## 2 Methods

### 2.1 Patients

The present investigation was a single‐center, retrospective, and observational study. Eligible patients were 20 years of age or older. Of the 631 patients who underwent complete R0 or R1(cy+) resection and developed recurrence at our institution between October 2002 and November 2017, 442 had wild‐type EGFR, 168 had *EGFR* mutations, and 21 could not be traced. Of the 168 with *EGFR* mutations, 162 with common mutations were included in this analysis. The median observation time of PRS was 4.95 years (range, 0.82–13.25), and POS was 5.81 years (range, 2.84–16.71). The histological and cytological diagnoses were performed according to the WHO classification criteria [11]. All patients were staged based on the International Association for the Study of Lung Cancer (IASLC) TNM (tumor, node, metastasis) classification, seventh edition [12]. At our hospital, we discussed the chosen treatment strategy for oligo recurrence in each patient at a multidisciplinary conference. This study was conducted with the approval of the Institutional Review Board of the Shizuoka Cancer Center (IRB registration number; J2022‐133), and the opt‐out method was adopted for obtaining informed consent from the patients.

### 2.2 Postoperative Follow‐Up and Diagnosis of Recurrence

Follow‐up examinations included a physical examination, hematological examination, and chest radiography. Chest and abdominal computed tomography (CT) were performed every 6 months during the first 3 years, and CT and chest radiography were performed alternately every 6 months thereafter. When disease recurrence was suspected, brain magnetic resonance imaging, bone scintigraphy, and fluorodeoxyglucose–positron emission tomography were additionally performed. Recurrent NSCLC was diagnosed based on the results of physical examination and of the findings of diagnostic imaging of lesions consistent with recurrent disease. Second primary lung cancers were generally differentiated from intrapulmonary metastasis according to the definitions proposed by Martini and Melamed [13]. Finally, we determined if recurrence had occurred in a patient by consensus at a multidisciplinary conference. Histological confirmation of the diagnosis was obtained where clinically feasible. The date of recurrence was defined as the date of radiological or histological confirmation or that of recognition of recurrent disease at the multidisciplinary conference in cases diagnosed based on clinical evidence. Oligo recurrence was defined as distant metastasis limited to 1–3 sites [14]. In this study, only the first recurrence after surgery was evaluated. Mediastinal lymph nodes were considered as one site, regardless of the number of lymph nodes affected. In the R classification, R0 was defined as no residual tumor and R1 as a microscopic residual tumor. If the pleural fluid was positive, it was designated R1(cy+). In this study, complete resection was defined as an R0 or R1(cy+) resection. Characterization of the *EGFR* mutations was outsourced to a commercial clinical laboratory.

### 2.3 Statistical Analyses

PRS was defined as the time from the date of the first confirmed recurrence to death or last confirmed survival. POS was defined as the time from the date of surgery to death from any cause or last confirmed survival. In the ADAURA trial, the allowed interval between surgery and randomization was 10 weeks in patients who did not receive adjuvant chemotherapy, and 26 weeks in those who received adjuvant chemotherapy. The probability of survival was estimated using the Kaplan–Meier method. Prognostic factors for PRS among the covariates at recurrence were identified using Cox proportional hazards analysis. The prognostic significances of all the variables were measured by calculating the adjusted HR with 95% CI. Logistic regression analysis was performed to identify significant background characteristics of the patients associated with bone or CNS metastases at recurrence. The variables were defined as factors reported to have a poor prognosis for OS in PR NSCLC [15, 16]. Eastern Cooperative Oncology Group Performance Status was excluded because all cases were 0 (51%) or 1 (49%), and this was, therefore, not considered to have an impact on prognosis. *p* values less than 0.05 were considered as statistically significant. All analyses were carried out using EZR version 1.60 (Saitama Medical Center, Jichi Medical University, Saitama, Japan) [17].

## 3 Results

### 3.1 Patient Characteristics

**FIGURE 1:**
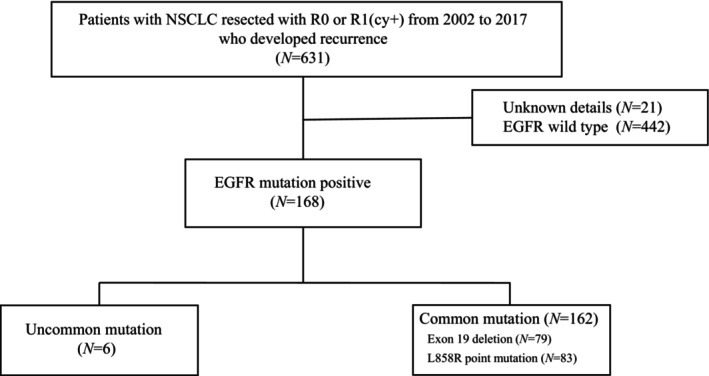
Flow chart of patient screening and enrollment.

A flow diagram of patient enrollment for this analysis is shown in Figure 1. The baseline characteristics of the patients are summarized in Table 1. The median age at recurrence was 70 years (range, 37–88), and the median time from sample collection to *EGFR* gene testing was 370 days (range, 0–2899). The majority of patients were female (62.3%) and nonsmokers (58%). The predominant histopathological type of NSCLC was adenocarcinoma (95%); lobectomy was the most commonly used resection procedure (90.1%). Wedge resection was performed in six patients owing to technical difficulties caused by severe adhesions in one patient and the presence of double cancers in the remaining five patients. The pathological stage was Stage I in 37.7%, Stage II in 25.9%, and Stage III in 36.4% of the patients. None of the patients had received neoadjuvant chemotherapy; however, 82 patients (50%) had received postoperative adjuvant chemotherapy. Eight patients had received investigational drugs as adjuvant chemotherapy, excluding the ADAURA cases. The residual lesions followed R0 resection in 132 patients and R1 resection in 30 patients. All R1 patients were evaluated as R1(cy+).

**TABLE 1:** Patient characteristics (*N* = 162).

	*N* (%)
Median age at recurrence, years (range)	70 (37–88)
Median time to EGFR gene testing, days (range)	370 (0–2899)
Median observation time, years (range)	4.95 (0.82–13.25)
Sex	
Male	61 (37.7)
Female	101 (62.3)
Smoking status	
Never smoker	94 (58.0)
Current or former smoker	68 (42.0)
Lung cancer resection type	
Lobectomy	146 (90.1)
Segmentectomy	6 (3.7)
Pneumonectomy	4 (2.5)
Wedge resection	6 (3.7)
Histology	
Adenocarcinoma	154 (95.0)
Adenosquamous carcinoma	5 (3.1)
Combined LCNEC	1 (0.63)
Large cell carcinoma	1 (0.63)
Pleomorphic carcinoma	1 (0.63)
EGFR genotype	
Exon 19 deletion/L858R point mutation	79 (48.8)/83 (51.2)
Residual lesion	
R0/R0 (un)/R1 (cy+)	99 (61.1)/33 (20.4)/30 (18.5)
Pathological stage	
I/II/III	61 (37.7)/42 (25.9)/59 (36.4)
Neoadjuvant chemotherapy	
Yes/no	0 (0)/162 (100)
Adjuvant chemotherapy	
Yes/no	82 (50.6)/80 (49.4)
UFT	22
CDDP regimen	49
CBDCA regimen	3
Investigational drugs	8

### 3.2 Pattern of First Recurrence and Initial Treatment

The diagnosis of recurrence was confirmed through histological examination in 27 patients and by radiological imaging in 135 patients; the diagnosis was not relied on clinical examination alone for any patients. Oligo recurrence occurred in 67 patients (41.4%) and non‐oligo recurrence in 95 patients (58.6%). Bone metastasis was observed as the first recurrence in 33 patients (spine, 16; other bone sites, 17), of whom 21 patients exhibited metastasis at other sites; CNS metastasis was observed in 39 patients (single site, 15; multiple sites, 21; meningitis, 3) and was associated with metastasis at other sites in 21 of these patients (Table 2).

**TABLE 2:** First recurrence sites and pattern of recurrence.

	*N* (%)
Diagnostics of recurrence	
Clinical assessment	0 (0)
Histological assessment	27 (16.7)
Imaging assessment	135 (83.3)
First recurrence sites	
Bone metastasis	33 (20.4)
Vertebra	16
Others	17
Involving other metastatic sites	21
CNS	35 (21.6)
Single brain metastasis	15
Multiple brain metastasis	17
Meningitis	3
Involving other metastatic sites	16
Pattern of recurrence	
Oligo recurrence	67 (41.4)
Non‐oligo recurrence	95 (58.6)

Abbreviation: CNS, central nervous system.

EGFR–TKIs were used (any treatment line) after recurrence in 155 patients (95.7%), in 61 of 67 patients with oligo recurrence, and in 94 of 95 patients with non‐oligo recurrence. TKIs were used as the first‐line therapy in 122 patients (75.3%), as the second‐line therapy in 23 patients (14.2%), as the third‐line therapy in 6 patients (3.7%), as the fourth‐line therapy in 1 patient (0.6%), and as the fifth‐line therapy in 2 patients (1.2%). The TKI used was gefitinib in 106 patients, erlotinib in 56 patients, osimertinib in 48 patients, and afatinib in 3 patients. Osimertinib was used as first‐line therapy in 23 patients, as the second‐line therapy in 9 patients, and as the third‐ or subsequent‐line therapy in 16 patients.

### 3.3 Survival/Regression Analysis

**FIGURE 2:**
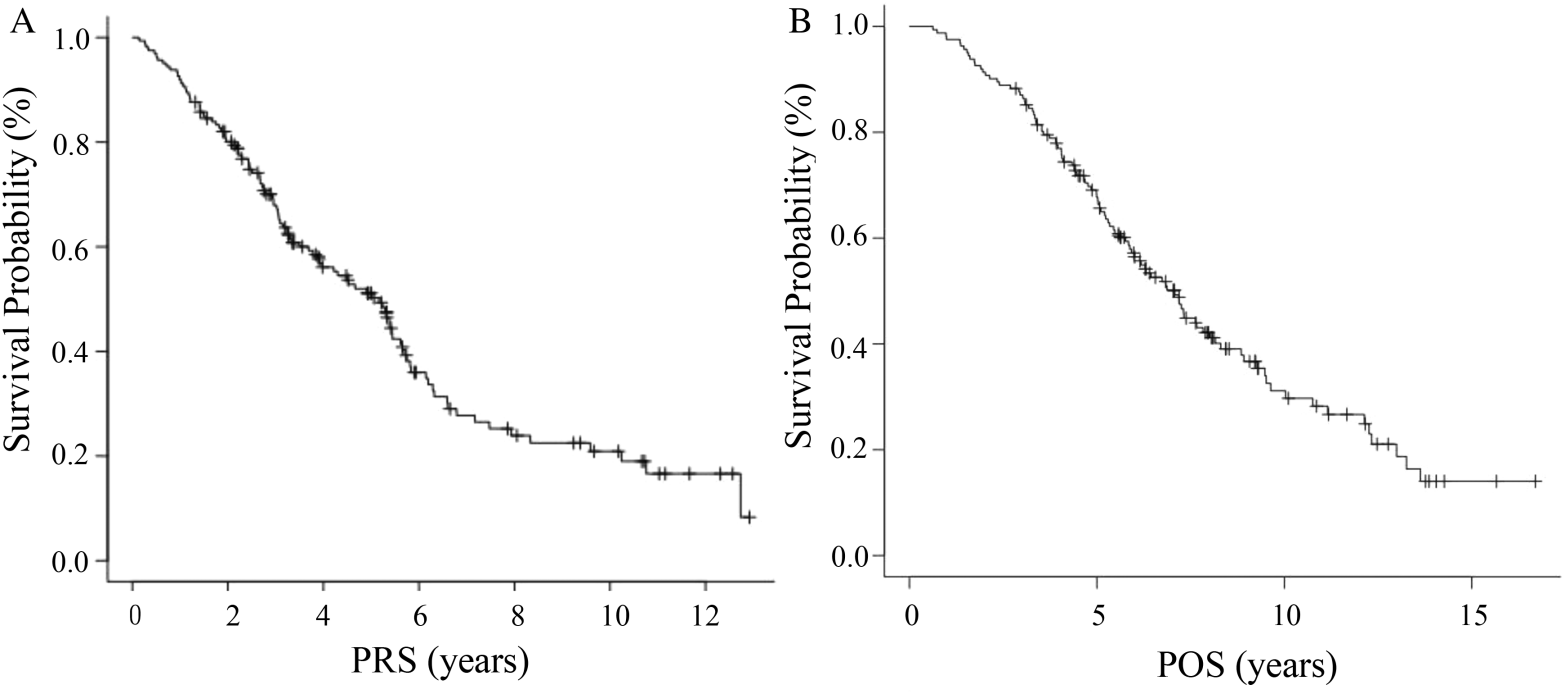
(A) Kaplan–Meier analysis of post‐recurrence overall survival; (B) Kaplan–Meier analysis of postoperative overall survival.

Survival analysis was performed on the 162 NSCLC patients with common *EGFR* mutations. The median PRS was 5.17 years (95% CI, 3.90–5.61) (Figure 2A). The median POS was 7.07 years (95% CI, 5.88–8.01) (Figure 2B), and the 5‐year survival rate postsurgery was 67.7% (95% CI, 59.8–74.4). The median PRS adjusted according to the ADAURA trial for the starting point was 6.70 years (95% CI, 5.43–7.66), and the 5‐year survival rate was 61.6% (95% CI, 53.4–68.7). The median observation time of PRS was 4.95 years (range, 0.82–13.25), and POS was 5.81 years (range, 2.84–16.71). The clinical variables identified by univariate analysis as being associated with significantly unfavorable survival were the presence of bone metastasis (median PRS: 5.33 vs. 2.43 years; *p* < 0.05), the presence of CNS metastasis (median PRS: 5.39 vs. 3.05 years; *p* < 0.05), and male sex (median PRS: 5.39 vs. 3.32 years; *p* < 0.05). Multivariate analysis identified the presence of bone metastasis (HR, 2.01; 95% CI, 1.23–3.28; *p* < 0.05) and CNS metastasis (HR, 1.84; 95% CI, 1.14–2.99; *p* < 0.05) as being independent unfavorable prognostic factors. No significant association was found with the pattern of recurrence (HR, 1.01; 95% CI, 0.67–1.54; *p* = 0.95) (Table 3). With regard to the factors associated with the presence of bone metastasis and CNS metastasis in this study population, there was a significant association between the presence of bone metastasis and male sex (OR, 0.14; 95% CI, 0.04–0.46; *p* < 0.05), and a trend toward an association was observed between the presence of CNS metastasis and male sex (OR, 0.35; 95% CI, 0.12–1.05; *p* = 0.06).

**TABLE 3:** Variables associated with post‐recurrence overall survival among 162 patients.

Co‐variable	No.	Univariate analysis		Multivariate analysis			
		median PRS (years)	*p*	Variate	HR	95% CI	*p*
Median age at recurrence							
<75	117	5.39					
≦75	45	4.53	0.10				
Sex							
Male	61	3.32		Male	0.89	0.51–1.58	0.70
Female	101	5.39	<0.05				
Smoking status							
Yes	68	3.69		Yes	1.44	0.82–2.52	0.20
No	94	5.39	0.09				
EGFR mutation							
Ex19 deletion	79	5.30					
L858R	83	3.90	0.30	L858R	1.07	0.71–1.61	0.76
Pattern of recurrence							
Oligo	67	5.24		Oligo	1.01	0.67–1.54	0.95
Non‐oligo	95	5.17	0.91				
Bone metastasis							
Yes	33	2.43		Yes	2.01	1.23–3.28	<0.05
No	129	5.33	<0.05				
CNS metastasis							
Yes	35	3.05		Yes	1.84	1.14–2.98	<0.05
No	127	5.39	<0.05				

## 4 Discussion

An important finding of this study was that the presence of bone and/or CNS metastases at recurrence was predictive of a poor prognosis in *EGFR*‐positive NSCLC patients with PR. Furthermore, the pattern of recurrence was not identified as exerting any significant influence on the prognosis.

In NSCLC patients with PR, the presence or absence of *EGFR* mutation, the number and sites of recurrence (intra/extra thoracic), and the presence/absence of CNS metastasis have been well established as prognostic factors [15, 16], although opinions conflict regarding the influence of bone metastases at recurrence on the prognosis [8, 9]. While the presence of bone metastasis at recurrence is reported as a poor prognostic factor in terms of both the PFS and OS in patients with advanced *EGFR*‐positive NSCLC [18], no reports are available on the prognostic impact of bone metastasis in *EGFR*‐positive NSCLC patients with PR. According to a previous report, the reason for the poor prognosis associated with bone metastasis in patients with advanced *EGFR*‐positive NSCLC is the presence, at a high frequency (72.7%), of multiple (three or more) distant metastases in these patients [18]. In the present study, among the 33 patients with PR, including those with bone metastasis, 12 (36.4%) had bone metastases only and 21 (63.6%) had other distant metastases in addition, showing an increased tendency for the presence of other distant metastases in patients with bone metastasis. This finding was consistent with the aforementioned report [18], although the number of coexistent metastases differed between the two studies. The number of patients who survived for more than 10 years after the diagnosis of recurrence was 20 (12.3%), including 8 patients with pathological Stage I disease, 4 with pathological Stage II disease, and 8 with pathological Stage III disease. Only 1 of these patients had bone metastasis, 2 had CNS metastasis (in 1 site of the CNS in 1 patient and in 2 sites in the other patient); about half of these patients (11 patients) had oligo recurrence.

Numerous studies have reported the relatively high risk of bone and CNS metastases in NSCLC patients with PR [19, 20]. According to one previous report, women are less likely to develop bone metastasis [19]. In addition, associations of preoperative carcinoembryonic antigen levels (>5 ng/mL) and positive pathologic lymph nodes with the development of bone metastasis and of poorly differentiated tumor and positive pathologic lymph nodes with the development of CNS metastasis have been reported [21]. Bone metastasis and male sex are thought to be associated regardless of the presence/absence of *EGFR* gene mutations; however, further investigation of the tumor markers and pathological findings and the risk of bone and CNS metastases in postoperative *EGFR*‐positive NSCLC patients is needed. In the ADAURA trial, a subgroup analysis showed a trend toward worse OS in male patients (HR, 0.62; 95% CI, 0.33–1.13) compared with that in female patients (HR, 0.41; 95% CI, 0.25–0.66) [6]. Similarly, in the present study, male sex was associated with the presence of bone and CNS metastases. As such, new treatment strategies are needed for patients with bone and CNS metastases at first recurrence.

Among the NSCLC patients with PR, the PRS was higher in the oligo recurrence group than in the non‐oligo recurrence group, and local therapy in the oligo group improved the PRS further [10]. However, local therapy has been reported to exert no influence on the PRS in *EGFR*‐positive NSCLC patients with PR, as TKIs are very effective in this patient group [22]. In the present study, 52 patients (78%) in the oligo recurrence group received local therapy, such as chemoradiotherapy, radiotherapy, or resection, but neither the treatment strategy used nor the pattern of recurrence had any influence on the prognosis, as previously reported [22]. Since the number of patients taking osimertinib developing recurrence is expected to increase, further studies are needed to develop effective treatments for postoperative NSCLC patients with bone/CNS metastasis at first recurrence. Inomata et al. reported that when bone metastases worsen in patients with *EGFR*‐positive NSCLC during EGFR–TKI treatment, the effect of the addition of radiotherapy while continuing EGFR–TKI results in PFS after relapse is comparable to that of subsequent chemotherapy. This finding suggests that the combination of continued EGFR–TKI and radiotherapy may be beneficial in patients with PR with bone metastases [23]. In contrast, Thomas et al. reported no difference in time to progression for *EGFR*‐positive NSCLC with CNS metastases when EGFR–TKI was combined with radiotherapy compared with EGFR–TKI alone; thus, the combination of radiotherapy might not be beneficial in CNS metastatic recurrence [24]. A randomized controlled Phase III trial comparing systemic versus local therapy for postoperative oligo recurrence in NSCLC patients is currently being planned (JCOG2108).

The reported PRS in *EGFR*‐positive NSCLC patients with PR treated with TKIs is 4.08 years [9], which is shorter than that determined in the present study. This could be attributable to the larger number of patients treated with osimertinib in this study. In both the osimertinib and placebo arms, the 5‐year survival rates were higher in the ADAURA trial than in this study. This result could be explained by the fact that 79 (38.5%) of the 205 patients in the placebo group who showed disease relapse in the ADAURA trial received osimertinib as the primary treatment after recurrence [6], compared to the 23 (14.2%) patients in the present study; in addition, we only enrolled patients with PR in this study. In the 23 patients in this study who received osimertinib as the first‐line therapy after recurrence, the PRS was still NA at the time of writing (95% CI, NA) and the 5‐year PRS was 100% (95% CI, NA). This result lends support to the rationale of using osimertinib as the first‐line therapy after PR. Although the ADAURA trial demonstrated that osimertinib treatment is associated with a reduced incidence of bone and CNS metastases [25], some patients still hesitate to take adjuvant osimertinib treatment because of the reported adverse effects. In the ADAURA trial, 36 patients in the osimertinib group developed adverse events; in 30 of these, the treatment was discontinued at the patient's request [5]. We consider that the findings of our present study are useful for patients considering resuming osimertinib.

This study had certain limitations. First, it was a retrospective study conducted at a single institution, and the possibility of selection bias cannot be ruled out. Second, histological confirmation was not obtained at recurrence in all patients. Third, we did not compare the prognostic factors with those in NSCLC patients with wild‐type *EGFR*. Fourth, osimertinib was not used in all patients. The FLAURA trial showed that osimertinib prevented progression of CNS metastases significantly more effectively than first‐ or second‐generation TKIs in advanced *EGFR*‐positive NSCLC patients [26]. In a population receiving osimertinib at a higher rate than that in the present study for PR, CNS metastases have previously been reported to have no prognostic impact [27]. However, that study focuses on patients who underwent radical resection for lung adenocarcinoma between 2015 and 2018, which may differ from the patient population of the present study, who underwent complete resection between 2002 and 2017. Furthermore, in the previous study, 46% of the cases received osimertinib, which is higher than the 14% in our study population. In addition, the ADAURA trial reported that osimertinib treatment yielded better CNS DFS in patients with Stages II–IIIA disease (HR, 0.24; 95% CI, 0.14–0.42) and a reduced incidence of bone metastasis (13 vs. 32 patients) [25]. Since the number of patients treated with osimertinib as adjuvant therapy is expected to increase, the poor prognosis observed in this study population may decrease, and the poor prognostic factors at first recurrence may differ from those in this study.

In countries where osimertinib is not approved for adjuvant therapy after PR with EGFR–TKI administration, the results of this study demonstrating its PRS and POS could help visualize future prospects. In countries where osimertinib is approved, these findings could assist in determining the timing of osimertinib initiation based on clinic visit frequency and cost considerations. In addition, the data on the median PRS and median POS of EGFR–TKI for PR may assist in selecting a treatment strategy for patients in countries where the use of osimertinib as postoperative adjuvant therapy is not permitted or for patients who refuse to take osimertinib as adjuvant therapy. Bone metastasis and CNS metastasis were identified as predictors of a poor prognosis in patients with *EGFR*‐positive NSCLC with PR. Thus, more effective treatment strategies need to be developed for EGFR‐positive NSCLC patients presenting with bone or CNS metastasis at first recurrence.

## Author Contributions

**Meiko Morita:** conceptualization, formal analysis, methodology, writing – review and editing. **Akira Ono:** project administration, supervision, writing – review and editing. **Motoki Sekikawa:** writing – review and editing. **Kosei Doshita:** writing – review and editing. **Keita Miura:** writing – review and editing. **Hiroaki Kodama:** writing – review and editing. **Michitoshi Yabe:** writing – review and editing. **Noboru Morikawa:** writing – review and editing. **Yuko Iida:** writing – review and editing. **Nobuaki Mamesaya:** writing – review and editing. **Haruki Kobayashi:** writing – review and editing. **Ryo Ko:** writing – review and editing. **Kazushige Wakuda:** writing – review and editing. **Hirotsugu Kenmotsu:** writing – review and editing. **Tateaki Naito:** writing – review and editing. **Haruyasu Murakami:** writing – review and editing. **Mitsuhiro Isaka:** writing – review and editing. **Yasuhisa Ohde:** writing – review and editing. **Toshiaki Takahashi:** writing – review and editing.

## Ethics Statement

All procedures performed in the human participants were in accordance with the ethical standards of the institutional and/or national research committee and with the 1964 Helsinki declaration and its later amendments or comparable ethical standards.

## Consent

We applied an opt‐out method to obtain informed consent for this study by posting a document about the study. The document was approved by the institutional ethics review board of Shizuoka Cancer Center (IRB number: J2022‐133‐2022‐1‐3).

## Conflicts of Interest

The authors declare no conflicts of interest.

## Data Availability

The datasets used and/or analyzed during the current study are available from the corresponding author on reasonable request.
